# Problem‐solving strategies used in anatomical multiple‐choice questions

**DOI:** 10.1002/hsr2.209

**Published:** 2020-12-03

**Authors:** Klodiana Kolomitro, Leslie W. MacKenzie, Mackenzie Lockridge, Diandra Clohosey

**Affiliations:** ^1^ Office of Professional Development and Educational Scholarship Faculty of Health Sciences, Queen's University Kingston Ontario Canada; ^2^ Department of Biomedical and Molecular Sciences Queen's University Kingston Ontario Canada

**Keywords:** assessment, higher order thinking, multiple‐choice questions, think‐aloud

## Abstract

**Background and Aims:**

Multiple‐choice questions (MCQ) in the anatomical sciences are often perceived to be targeting recall of facts and regurgitation of trivial details. Moving away from this assumption requires the design of purposeful multiple‐choice questions that focus on higher‐order cognitive functions as opposed to rote memorization. In order to develop such questions, it was important to first understand the strategies that students use in solving multiple‐choice questions. Using the think‐aloud protocol, this study seeks to understand strategies students use in solving multiple‐choice questions. Specifically, it seeks to uncover patterns in the reasoning process and tactics used when solving higher and lower order MCQ in anatomy. The research also provides insights onto how these strategies influence the student's probability of answering questions correctly.

**Methods:**

Multiple‐choice questions were created at three levels of cognitive functioning based on the ideas, connections, extensions (ICE) learning framework. The think‐aloud protocol was used to unravel problem‐solving strategies used by 92 undergraduate anatomy students as they solved multiple‐choice questions.

**Results:**

Sixteen strategies were identified through the oral and written think‐alouds that students used to solve MCQ. Eleven of these have been described and supported by the literature, while the rest were utilized by our students when solving MCQ in anatomy. Domain‐specific strategies of visualizing and recalling had the highest use. Personal connection was a strategy that allowed students to achieve success in all ICE levels in the oral think‐alouds and in the I and E levels in the written think‐alouds.

**Conclusions:**

This research argues that it is upon us as educators to make learning visible to our students, specifically through the use of think‐alouds. It also raises awareness that when educators facilitate the process of students making personal connections, it aids students in new knowledge being integrated effectively and retrieved accurately.

## 1 INTRODUCTION

Assessment is a central motivator for students and can influence the way they approach the learning of course material. As Boud noted, “What and how students choose to learn is, in large part, influenced by what and how we choose to assess.”^1^ Therefore, educators need to ensure that the structure and focus of our assessment plan do not inadvertently deter students from meaningful learning. In anatomy, multiple‐choice exams, although not the only means of evaluation, are a signature assessment strategy,^2^ popular with instructors and frequently considered necessary in the discipline. Students tend to associate multiple‐choice exam format with memorization and may not see the need to modify their study approach to think critically about the material.^3^ This often happens when multiple‐choice questions (MCQs) are not purposefully designed nor aligned with the level of learning that is intended. The ideas, connections, extensions (ICE) model^4^, ^5^ offers a useful framework in designing assessments that target various levels of cognitive function. The ICE model includes three components: ideas, connections, and extensions, which represent various frames of learning. Ideas are the fundamental, discrete pieces of information that make up the building blocks of learning. Connections are the relationships that students can form among discrete ideas,and connecting new concepts to prior knowledge. Extensions constitute creating new learning and applying knowledge to completely new and novel situations.

There is a gap in the literature on domain general and specific strategies students use in solving MCQs. Domain general strategies can be observed to be executed across multiple domains and are not dependent on content knowledge.^6^, ^7^ On the contrary, domain‐specific strategies are focused on content knowledge and depend on the domain the task is in.^6,7^ Using the think‐aloud protocol, this study seeks to understand strategies students use in anatomical education to answer both lower order and higher order MCQ and provides insight onto how these strategies influence the student's probability of answering questions correctly.

## 2 METHODS

### 2.1 MCQ Development

This research was granted clearance by Queen's University Health Sciences and Affiliated Teaching Hospitals Research Ethics Board (DBMS‐068‐17), and participants provided informed consent. The overarching research questions that informed our research design were: (a) What strategies are used by undergraduate anatomy students when solving multiple‐choice questions? (b) How do these strategies influence the student's probability of answering questions correctly?

Multiple‐choice questions were designed to be included in this study. The questions were developed using the ICE framework. Two co‐authors, who are also anatomy instructors, independently aligned the MCQs with the ICE model. Initially, the authors developed eight questions, but only six were included in this study based on the authors' agreement of the MCQs with the appropriate level in the ICE framework. A summary of the multiple‐choice questions and the co‐authors consensus is provided in Table 1.

**TABLE 1:** Multiple‐choice questions used in the think‐alouds and their alignment with I, C, or E level

Correct written answer*	Question	Level in ICE
The trochlea is part of which of the following bones? a. Scapula b. Ulna c. Radius * d. Humerus		I
Fissures divide the lungs into: a. Lobules * b. Lobes c. Alveolar sacs d. Segments		I
A 20‐year‐old patient cannot abduct and medially rotate the thigh while running and climbing. Which of the following muscles is most likely damaged? a. Semimembranosus b. Sartorius *c. Rectus* femoris * d. Tensor fasciae latae		C
In a given muscle fiber at rest, the length of the “I” band is 1.0 um and the “A” band is 1.5 um. What is the length of the sarcomere? a. 1.5 um b. 2.0 um * c. 2.5 um d. 3.5 um		C
Kyphosis affects the structure of vertebrae causing forward rounding and abnormal curvature of the spine. Regarding the anatomy of the spine and associated axial skeleton, what may be the functional implications of this bony disorder? a. shorter stature b. change in shape of the thoracic cavity c. odd shaped stomach * d. two of the above options		E
Why are the ligaments of the knee more prone to injury when the foot is planted (ie, on the ground) with the leg extended rather than flexed?” a. ligaments are loose * b. ligaments are tight c. they play no role in the stability of the knee d. the knee is easily moved under these conditions		E

### 2.2 Study participants

This study utilized purposeful sampling and “intentionally select[ed] individuals and sites to learn or understand the central phenomenon.”^8^ We recruited participants from a second‐year undergraduate anatomy course offered at Queen's University, a mid‐sized research‐intensive Canadian university. The participants in this study accurately reflect our population of interest, which is undergraduate health sciences students. Participation in this research was entirely voluntary and not linked with any assessments in the course. It also did not impact students' overall academic standing or relationship with the institution. As one of the authors was the course instructor, recruitment was done by another co‐author. The selected course is a core course for students in the Life Sciences degree program, and it is designed to introduce general principles of the structure and function of human body systems. One of the co‐authors was invited to the classroom to provide information about the research study and give students an opportunity to ask questions.

### 2.3 Data collection

In this study, the think‐aloud protocol was used to study students' thought processes when responding to MCQ. The think‐aloud approach^9^ provides an opportunity to obtain rich, deep, and descriptive data from the participant's experiences, perceptions, and meanings as it requires participants to verbalize their thought process as they solve a task. The focus is on the cognitive processes, rather than the final product, with the goal of making these processes as explicit as possible during task performance. Thirteen students expressed interest in participating in the oral think‐alouds; however, due to scheduling conflicts only 10 students were included in this stage. Ten oral think‐alouds, followed by 82 written think‐alouds administered through a questionnaire, were used as the source of gathering data. The oral think‐alouds helped to validate the questions to be used in the written think‐aloud. One‐on‐one interviews were scheduled with those students who agreed to participate in the oral think‐alouds. The interviews ranged from 40 to 60 minutes in length and were audio‐recorded and transcribed. Prior to each think‐aloud interview, a think‐aloud practice activity was designed and implemented to help students feel comfortable with this approach (Appendix) and model the depth of responses. In the written think‐alouds, students were asked about their use of strategies based on the level of question given to them. Both oral and written think‐alouds were utilized to reach data saturation.

### 2.4 Data analysis

The qualitative content analysis protocol^10^ was followed to identify operators that students utilized when working through those questions. These operators explained the predominant reasoning processes used by the students. We followed a hybrid approach to the analysis where we began with a set of a priori codes and then added to them as we analyzed the data inductively.^11^ The 11 strategies that were previously identified in the literature guided the development of the interview script. During a working meeting, two co‐authors selected one of the six problems and categorized several of the think‐alouds independently. When this process was complete, we discussed our categorizations including any disagreements. Once we reached agreement on all categories for a single multiple‐choice question, we then chose a subset of think‐alouds for the purpose of determining interrater reliability. If the interrater reliability was below an acceptable level (0.60 Kappa value), we started the process again following the same steps. This process helped us not only compare segments of data to each other but also to the identified categories to see whether the data were confirming or disconfirming the existing categories.^12^ Once there was more clarity in these procedures, we combined similar categories and refined our list.

## 3 RESULTS

### 3.1 Student strategies for MCQ

Sixteen strategies were identified through the oral and written think‐alouds that students used to solve MCQ (Table 2).

**TABLE 2:** The 16 strategies observed from the oral and written think‐alouds

Strategies	Description for the category
Keywords Comparing language of options Read aloud Asking a question Delaying Determining question type Correcting Recognizing/familiarity Adding information Checking Predicting Recalling Imitating Mnemonic Personal connection Visualizing	Picked out keywords from the question and focused on them Detect similarities or differences in the language of options and determine correctness Read the question and/or the options aloud After reading the question or options asked a question regarding the problem Consider one of the options and decide that it should not be eliminated, the quality of that option should be evaluated later, after the other options are considered Placing a label on the question in terms of type, for example, as memorization, application, etc. Pointing out that they had been thinking incorrectly about a problem, correct it Perceived a question or answer as correct or incorrect without a rationale or due to it being familiar to them Provided more information about one of the options, such as additional facts that were omitted or corrections to incorrect statements (ie, presented incorrectly to serve as distractors) Explained why an option is correct or incorrect by comparing the options with their knowledge or with the data provided in the problem Predicted what they expected the answer to be Retrieved basic facts or concepts from class, lab, notes, or the textbook, that is, declarative knowledge Imitating terms presented in the question, for example, moving Utilizing a mnemonic device when responding to a question Expressing a personal connection to a topic presented in the question Convert the written information to a visual or draw written information as a visual

Eleven of these strategies, categorized as domain general, have been already described and supported by the literature as procedures that learners frequently used in problem‐solving.^4^, ^5^, ^6^, ^7^ Strategies like checking, comparing, recalling, and predicting are associated with the ICE Framework.^4^, ^5^ Five domain‐specific additional MCQ solving strategies were identified, that were practiced by our students when solving MCQ in anatomy. These strategies were visualizing, mnemonic, imitating, personal connection, and recalling.

A strategy such as visualizing is more dependent on a domain as the nature of anatomy plays high importance to the location of human structures in the body and their directional relationships to other structures. Methods used for instruction and studying anatomy often utilize visual aids. Mnemonics and imitating were used as memory devices to help students remember and retrieve information. In mnemonics particularly, students utilized phrases, rhymes, and acronyms to help recall concepts when answering the questions. As an example, participant 2 used the mnemonic “dArk lIght” to remember that in a sarcomere, the A band is the dark region and I is the light region. Conversely, with imitating students use learned functions or movements of the human body from the course and utilized their own body to recreate an action or term that was stated in the question. In recalling, students used pattern recognition and relied on declarative knowledge to answer the multiple‐choice questions. Students highlighted anatomical structures that they recalled from attending the laboratory sessions or facts they remembered reading about in the anatomy lecture outlines. In personal connection, students interacted with the question and made connections between anatomy and their personal life. Participants were specifically thinking how anatomy was integrated in their everyday living, for example, as they played sports, went to the gym, or when they had an injury. When analyzing the question levels with the strategies, domain‐specific strategies increasingly were used from I to C to E level questions, with a total of 51 of the domain‐specific strategies utilized when solving the E‐level questions.

### 3.2 Associating strategies with question types

Another goal was to further explore in what types of questions (I, C, or E) were strategies used and which of those strategies allowed for correct answers more than 50% of the time (Table 3). Recalling and visualizing were the two domain‐specific strategies that were highly utilized across all levels of questions in both oral and written think‐alouds. Recalling leads to correct answers usually (greater than 70%) across all levels in the oral think‐alouds and in the I and E levels in the written think‐aloud question. Despite the frequent use of visualizing in the written think‐aloud participants, it resulted in a high likelihood (>50%) of getting the incorrect answer for question levels C and E. An example of using visualizing and recalling together is with participant 5's interview when answering the “kyphosis” question they stated, “First I tried to remember what kyphosis was, and then what maybe the function implications, I was trying to picture from my notes what it looks like.”

**TABLE 3:** Frequency of I, C, and E level questions for both oral and think‐aloud procedures. Frequencies lower than 15 were categorized as lower frequencies and everything above that number as higher frequencies

			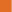 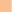	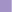 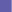					
			Lower frequencies	Higher frequencies					
Part A: Domain general procedures									

	Adding information	Checking	Recognizing/familiarity	Correcting	Predicting	Key words	Comparing language of options	Delaying	determining Question type	Read aloud		Asking a question			
I level questions	1	4	9	11	8	23	0	3	4	5		0			
C level questions	0	4	5	8	15	27	0	3	3	2		1			
E level questions	4	10	1	7	28	24	6	10	4	5		1			
Part B: Domain‐specific procedures															
	Imitating	Visualizing		Recalling	Mnemonic	Personal Connection									
I level questions	11	40		35	5	5									
C level questions	23	34		30	9	11									
E level questions	12	22		32	22	9									

Personal connection was another strategy that was highly utilized in both oral and written think‐alouds. During the oral think‐aloud, personal connection helped participants come to the correct answer 100% of the time. During the written think‐aloud, when personal connection was used in the levels of I and E, it had an influence of being successful (I‐100%, E‐62%) vs using a personal connection in the connecting level questions resulted in the incorrect score 81% of the time. An example of using personal connection is during participant 2's oral think‐aloud, they made a personal connection to help them come to an answer as noted by their statement, “Also at the beginning of the question, I thought about when I played sports to compare stable flexed position to unstable extended.”

## 4 DISCUSSION

The findings of this study clearly support other research related to problem‐solving strategies of health sciences students answering multiple choice questions. Past research highlights that students employ different strategies based on the cognitive level of the test items.^6^, ^7^ This study adds to the prevailing literature by identifying strategies utilized specifically in solving anatomical questions, which could help inform instructional methods and course design. It also offers insights and evidence into the importance of making learning visible and narrowing the gap between novice and expert thinking.

### 4.1 Making learning visible

The findings suggest that as students are solving more in‐depth level (C and E) questions, they use more strategies, although this is not necessarily helping them get the right answer. These students would benefit by being more selective of their use of strategies when solving MCQ. To help students be more selective, we as educators need to explicitly model our own mental processes so that students recognize how the experts take preexisting knowledge and their perspective of the problem, to sequentially manipulate the problem in order to generate a solution.^12^ Decoding the disciplines, a theory of pedagogy, highlights cognitive bottlenecks for students.^13^ These cognitive bottlenecks, such as activating prior learning or making connections, exist when educators are not explicitly displaying to students how they use their thinking in their fields.^13^ Educators may at times take for granted the knowledge they have, how they see the problems in the field, and the process they use to successfully solve this problem. Many of the mental steps taken by educators could be easily perceived, yet these steps are not translating well to our students. This supports the claim that the bridge between teaching and learning still needs to be refined.

### 4.2 Narrowing the gap between novice and expert thinking

It was seen in our findings that students consistently are not achieving success particularly in the question level of connecting. This may be due to the differences in knowledge organization between experts and novices. Experts' knowledge is organized in web‐like structures and connected intricately and in a meaningful way.^14^ Conversely, novice learners organize and connect learned knowledge in a more linear and superficial way, so when asked to access their knowledge, it is often based on loosely connected facts.^14^ Making connections more explicit for the students encourages engagement, supports deeper learning, intrinsically motivates, and allows for application of knowledge.^15^, ^16^ It also creates a sense of difficulty that is desirable for students.^17^

Students need to be given the opportunity not only to have guided instruction of strategies but also to practice these strategies. Think‐alouds can be applied by students to practice higher order cognitive thinking strategies for answering multiple‐choice questions. Students can monitor their reasoning process and apply specific strategies they feel will most efficiently and effectively result in a correct answer and promote deeper learning. Moreover, student think‐alouds can be assessed by educators to provide specific feedback that can pinpoint areas of struggle in the students' thinking.^12^ By providing a structure for students to build their connections around, it can help highlight to students where deeper connections can be made.

### 4.3 Limitations

As think‐aloud procedures are time consuming, there was no opportunity to meet with the students beforehand and properly mentor them on “how to think‐aloud.” As a result, while several problem‐solving strategies were identified, there might have been other thoughts or procedures that were not captured if the students did not state those. Also, it was at times difficult in finding what is the “right” amount of probing. Yet, think‐alouds remain a powerful vehicle in making visible metacognitive processes that often remain hidden to both the participants and researcher. Another limitation relates to analyzing only six questions using think‐alouds. Hence, the conclusions of the authors must be treated with some level of caution. Further research exploring the relationship of strategy‐to‐question type would be beneficial in enhancing the transferability of our findings. Finally, it is worth noting that both oral and written think‐alouds were used in this research, and they have their own advantages and disadvantages. These limitations in the research design must be acknowledged; nevertheless, the study provides useful insights into understanding strategies that students use in solving multiple‐choice questions.

## 5 CONCLUSION

This research offered insights, through the think‐aloud protocol, into the mental processes and strategies students use when answering MCQs in anatomy. A total of 16 strategies were discovered to be employed by students, 11 of which correlated with previous literature. Domain‐specific strategies of visualizing and recalling had the highest use throughout both oral and written think‐alouds. Personal connection was a strategy that allowed students from the oral think‐aloud to achieve success in all levels, signifying that anatomical educators need to be aware that when students make personal connections it aids in new knowledge being integrated effectively and retrieved accurately. We argue for making learning visible to our students and supporting them in deconstructing their problem‐solving strategies.

## AUTHOR CONTRIBUTIONS

Conceptualization: Klodiana Kolomitro, Leslie W. MacKenzie, Diandra Clohosey

Investigation: Klodiana Kolomitro, Leslie W. MacKenzie, Mackenzie Lockridge, Diandra Clohosey

Methodology: Klodiana Kolomitro, Leslie W. MacKenzie, Mackenzie Lockridge, Diandra Clohosey

Writing ‐ Original Draft Preparation: Klodiana Kolomitro, Leslie W. MacKenzie, Mackenzie Lockridge, Diandra Clohosey

Writing ‐ Review and Editing: Klodiana Kolomitro, Leslie W. MacKenzie, Mackenzie Lockridge

  All authors have read and approved the final version of the manuscript.

  Klodiana Kolomitro has full access to all of the data in this study and takes complete responsibility for the integrity of the data and the accuracy of the data analysis.

## CONFLICT OF INTEREST

The authors declare no conflict of interest.

### TRANSPARENCY STATEMENT

Klodiana Kolomitro affirms that this manuscript is an honest, accurate, and transparent account of the study being reported; that no important aspects of the study have been omitted; and that any discrepancies from the study as planned (and, if relevant, registered) have been explained.

## Data Availability

The authors confirm that the data supporting the findings of this study are available within the article and in the appendix. If further raw data are desired, please make this request to the corresponding author.

## INTERVIEW SCRIPT A

Practice exercise: You open the door to your apartment and you need to get to put the milk you had just bought in the fridge. What are the steps you would take? This practice question is to help you get a feeling to the level of detail that we are looking for.

1. What did you think when you first saw the question? What do you think the question is asking?
2. Does it seem like a difficult question? What do not you understand about the question?
3. How would you go about answering it?
4. How does it fit in with what you already know?
5. What do you anticipate the answer to be?
6. What are the steps you are taking to review the multiple‐choice options?
7. What do you know about this topic?
8. What images/pictures you create in your mind connected to the words you are reading?
9. What kind of knowledge is required to answer this question?
10. How do you think these questions were different?
11. What are the steps needed to answer this question?
